# Language complexity of patient-physician chat communication on hypertension control: results of the cluster-randomised PIA study

**DOI:** 10.1186/s12916-025-04006-0

**Published:** 2025-03-24

**Authors:** Simon-Konstantin Thiem, Lucas Küppers, Benjamin Aretz, Arezoo Bozorgmehr, Arian Karimzadeh, Frauke Leupold, Birgitta Weltermann

**Affiliations:** Institute of General Practice and Family Medicine, University Hospital Bonn, Venusberg-Campus 1, Bonn, 53127 Germany

**Keywords:** Information communication technology, Chat analysis, Patient-physician communication, Language complexity, Flesch index, Hypertension, Digital care, Family medicine, General practice, mHealth

## Abstract

**Background:**

High language complexity impairs patients’ understanding and medical outcomes. While messengers accelerate communication, the language complexity of chats between patients and providers is poorly studied. This study analyses language complexity and communication characteristics of chat data from the PIA study, which significantly improved blood pressure control after 6 to 12 months.

**Methods:**

The cluster-randomised controlled PIA study enrolled 848 hypertension patients (412 intervention, 436 control) from 64 German general practices. The PIA technology enabled a secured communication of blood pressure readings, medication plans and messages. The chats were analysed regarding frequency, length, response time and content. Language complexity was measured using the Flesch index with seven levels from ‘hard’ to ‘very simple’. The study is registered in the German Clinical Trials Register (DRKS00012680).

**Results:**

In total, 4231 messages were sent between 24 general practitioners and 363 patients of the intervention arm between 09/20 and 09/21: 22% messages (*n* = 941) were automated (new medication plan or prescription available), while 78% were non-automated (*n* = 3290), with 41.1% of these messages originating from patients and 58.9% from practices. The average chat dialogue lasted 176.8 days (SD 9.8). Patients’ messages had a mean of 22.6 words (SD 22.6) compared to 16.8 (SD 19.4) by practices. Most messages (88.92%) from practices and 51.9% from patients addressed medication or treatments. Simple or very simple language was used in 90.5% of the messages both by patients and by physicians regardless of sociodemographic characteristics. BP improved with increased frequency of messages (*p* < 0.001).

**Conclusions:**

This communication showed a remarkably low language complexity by physicians and patients and better control with more messages. The results support the use of digital communication for topics such as chronic hypertension care.

**Trial registration:**

German Clinical Trials Register, DRKS00012680. Registered May 10th, 2019, https://www.drks.de/drks_web/setLocale_EN.do.

## Background

Effective patient-physician communication is the key to patient satisfaction, prevention of adverse events and desired health outcomes [1, 2]. However, communication can be impaired by low language proficiency on behalf of patients and high language complexity on behalf of providers. Divi et al. showed that people who did not speak English had a 19.6% higher chance of physical harm due to an in-hospital adverse event [3]. In a multi-centre Canadian study, language discordance was as a marker of disparity in stroke outcomes [4]. In addition, high language complexity by providers and in medical documents impairs patients’ understanding and outcomes [5, 6]. To decrease such risks and improve the inclusion of disadvantaged populations, simple language approaches and tools to evaluate language complexity such as the Flesch index [7] and the Flesch-Kincaid grade level [8] were developed. These indices use word and sentence lengths to describe the structural difficulty of a text with a scoring system. Applying the Flesch index, Gordejeva et al. showed a high prevalence of difficult-to-read language in Wikipedia texts written in English (96.9%; *n* = 6125), Russian (79.9%; *n* = 3313) and German (99.7%; *n* = 6022) across ICD-10 chapters [9]. The readability of four informed consent documents for COVID-19 vaccine trials exceeded grade 9 language complexity and scored lower than 60 in the formal reading ease metric, thus constituting difficult language [5].

Recently, emails, SMS, blogs and personal chats via messengers have influenced everyday contacts between patients and providers [10]. Uses span from simple practical information (e.g. scheduling appointments) to complex digital interventions for improving care processes and health outcomes, e.g. in fertility treatments [11], cancer and cancer aftercare [12–14], and HIV prevention [15]. Despite the widespread use of such digital communication, scientific studies of personal electronic chat communication between patients and physicians are rare. In a qualitative study, Stommel and van der Houwen compared 22 email and 15 synchronous chat dialogues between patients and psychological counsellor-trainees on anxiety or depression [16]. While patients tended to self-diagnose more often in chats, both patients and counsellors were more detailed and reflective in emails [16]. Aiming to understand how electronic communication is related to health outcomes, Heisey-Grove et al. developed a theory-based taxonomy to code technology-mediated communication and coded 18,309 messages of 1031 patients (with diabetes and/or hypertension) and 566 clinic staff (physicians and non-physician staff) from a large urban US medical centre. The initiating messages most frequently addressed information seeking (29%; of these: 71% seeking medical guidance), scheduling (28%), information sharing (23%) and prescription requests (23%). Addressing associations with health outcomes in a study of 1602 patients, the same researchers showed that patients who initiated threads had larger declines in HbA1c. Overall, results regarding health outcomes were mixed, e.g. patient-generated appreciation of providers was associated with higher HbA1c levels, while patients’ complaints were associated with lower systolic blood pressure (BP). However, these studies did not address language complexity. In 2013, Mirsky et al. performed a Flesch index analysis of 119 mails between 31 low-income patients and their primary care providers from a Californian clinic: 68% had a Flesch-Kincaid grade level corresponding to the conversational English of at least 8th and 9th grade, with no difference between providers and patients [17]. However, more in-depth research on language complexity in patient-provider emails and chats is lacking.

This study analysed data from the cluster-randomised PIA study (PC-supported case management of hypertensive patients to implement guideline-based hypertension therapy using a physician-defined and supervised, patient-specific therapeutic algorithm) which included a total of 848 patients and 64 general practices [18, 19]. In the intervention arm, the PIA information communication technology (PIA-ICT) enabled a secured communication of blood pressure (BP) readings, medication plans and chat messages between the patients’ PIA app and the practices. In comparison with the control group receiving usual care, the PIA intervention improved the BP control rate (practice BP < 140/90 mmHg) by 23.1 percentage points after 6 to 12 months. The high acceptance and usability of the PIA system is reflected in the frequent use of the PIA-ICT as well as the consistently positive evaluations from all users in the pilot and effectiveness studies. Patients, GPs and practice assistants rated the system as ‘very good’ on a five-point scale [18, 19]. Using a double-pseudonymised data set from the PIA study, the present study analysed the PIA chat messages regarding language complexity, chat contents and further communication characteristics. In addition, associations between chat frequencies and BP control were evaluated.

## Methods

### Study design and conduct

This study analysed chat dialogues from the intervention arm of the PIA study. All participants who sent or received at least one text message were included.

The PIA study was conducted as a cluster-randomised controlled trial in 64 German general practices from 09/2020 through 09/2021. The study protocol and the main results are published [18, 19]. In short, patients aged 40 to 79 years with uncontrolled blood pressure (≥ 140/90) and the need for at least one anti-hypertensive medication were included. All participants (physicians and patients) provided written informed consent. The enrolled practices were randomly allocated (1:1) to the intervention group (using the PIA-ICT) or the control group (usual care). Ethical approval was obtained from the Ethics Committee of the Medical Faculty of the University of Bonn (reference number: 156/18, date of approval: 02/08/18). In addition, the data protection officer of the University Clinic Bonn approved the study. All data were collected in compliance with European data protection laws. The PIA study was registered in the German Clinical Trials Register (DRKS00012680).

### PIA intervention

The intervention group received the complex PIA intervention: the PIA-ICT with the application (PIA app) for patients and the practice management centre (PIA-PrMC) for practices. Both target groups received an educational program (PIA education). In addition, practices received information on the recruitment process and on how to obtain standardised BP measurements. The PIA app offered the following functions:

- Chat communication between patients and physicians
- Prescription requests
- New medication plans as adjusted by the physicians
- Transmission of the patients’ BP readings (systolic, diastolic, heart rate) associated with indicators of activities and subjective well-being (smileys) to the practice

Additional information on the PIA app, the PIA-PrMC and the PIA education are published in the study protocol [18].

The care process with the PIA-ICT consisted of the following steps: patients transmitted their resting blood pressure. The practice monitored the transmitted BP readings at least every 7 days and adjusted the medication accordingly. This process was repeated until the patient was within the target range. The GP assigned individual measurement intervals once the patient’s BP was controlled. Patients and practices communicated via the chat system. Safety assessments and adverse events are described in the PIA study protocol [18].

### Outcomes

The primary outcome of this study was the language complexity of patients’ and physicians’ chat messages. Language complexity was measured using the Flesch index with seven levels ranging from ‘hard’ to ‘very simple’ and was further assessed with the German version of the Flesch Reading Ease test. This test evaluates the readability of a written text by counting the number of words and syllables in a sentence. Conceptually, the Flesch index evaluates language complexity on a quantitative level on the underlying assumption that sentences containing more words and words containing more syllables are more difficult to comprehend. The ASL (Average Sentence Length) is the number of words divided by the number of sentences in a text. The ASW (Average Number of Syllables per Word) is the number of syllables in a text divided by the number of words in a text. Using the following formula for the Flesch index in German [20] (Flesch value = 180 − ASL − (58.5 * ASW)), the Flesch score is categorised as follows: < 30—hard, 30– < 50—difficult, 50– < 60—fairly difficult, 60– < 70—plain German, 70– < 80—fairly easy, 80– < 90—easy, 90–100—very easy. The German Flesch index differs slightly from the English version in two aspects: The weighting factor for the ASL is set at 1 (English: 1.015) and the ASW is lowered to 58.5 (English: 84.6) because the German language has inherently longer words [20]. The patients’ and practices’ mean Flesch values were correlated with the respective sociodemographic characteristics of patients and physicians.

The following secondary outcomes were assessed:

1. Chat characteristics (total and per patient): number of messages, number of patients with at least one message, number of automatically generated messages (new medication plan, prescription ready for pick-up), duration of conversation first to last message (in days), length of messages from practices to patients (words, characters), length of messages from patients to practices (words, characters), period between messages (practice, patient) (in days).
2. Frequency of words used (per patient, per message) including salutations and goodbye phrases: The words, predominantly in German, were sorted manually into topic categories. Specifics of the German language were respected: e.g. conjugations like ‘impfen’ (vaccinating) instead of ‘Impfung’ (vaccination), or compound words like ‘Medikationsplan’ (medication plan), which were analysed by word stem.
3. Content of the chat communication: A coding matrix was developed using deductive and inductive approaches based on the literature including the above-mentioned taxonomy by Heisey-Grove [21–27]. First, these codes were exploratively applied to 40 randomly selected messages by the three coders (coder A, coder B, coder C). Discrepancies were discussed between the coders and redefined. This procedure was repeated with another set of 30 messages and the code matrix was then finalised. Subsequently, one researcher (S.T.) coded all messages and the other two researchers (L.K., B.A.) each coded half of the messages. Differences in coding were discussed until a consensus was reached. The coding system for the messages comprised the following headings and subheadings:
  - Messages from practices to patients:
    Medications or treatments: update on BP treatment, measurements, medication; request for (and inquiries about) BP measurements; BP medication change; inquiries about patient’s well-being; in-office consultation; update on other treatments and/or medication; inquiries about vital signs, symptoms, problems or diseases; phone call request; measures against other symptoms, problems or diseases
    Study conduct: messages on further procedures, (chat) technology
    Risk factors: nutrition, psychosocial problems, life issues, physical activity
    Administration: appointment request, prescription refill request
    Other: confirmation, thanks, other
  - Messages from patients to practices:
    Medications or treatments: other medication and/or treatments; symptoms, problems or diseases; BP medication; disclose BP; inquiries on further procedures; measure BP; prescription change; in-office consultation
    Study conduct: (chat) technology; inquiries on further procedures
    Risk factors: nutrition; psychosocial problems; life issues; physical activity
    Administration: scheduling request; phone call request; referral request; prescription refill
    Other: confirmation or thanks, other notification, other

The coding process was documented digitally using a self-written program in Python.

In addition, the following baseline information on sociodemographic and medical characteristics was analysed:

- Patient characteristics: age (years), sex assigned at birth (male, female), relationship status (married/living with partner, divorced/separated, widowed, single), education (completed 13th grade (Abitur), 12th, 10th or 9th grade, no degree, other), professional status (employed, early or regular retirement, job-seeking, homemaker, unemployed), self-assessment of health (bad, not good, good, very good, excellent).
- Physician characteristics: sex assigned at birth (male, female), practice type (single, group practice), years since licenced for statutory health insurance, affinity for information technology (composite mean score of the 9 items from the Affinity for Technology Interaction (ATI) scale ranging from 1 [low technical affinity] to 6 [high technical affinity]) [28].

Data from the PIA-ICT from baseline to follow-up contained the following information:

- Home BP measurements: Patients were asked to perform standardised resting blood pressure (BP) measurements at home (twice in the morning, twice in the evening, each with a 1-min interval). Once patients’ BP was controlled (BP < 135/85 mmHg) measurement frequencies were adjusted. The patients transmitted their well-being using one of five smiley icons with the BP measurements. The five smiley options were labelled with a value between 1 (bad) and 5 (good). We calculated the average time between a blood pressure measurement/smiley and the closest message.
- Chat communication: The dates, times and content of the chat communication between the physicians and the patients were documented.

### Statistical analysis

#### Data set preparation

The chat data were exported from the PIA-ICT as pseudonymised text files with study IDs. If data contained a patient’s or physician’s name within the chat, these were removed. This cleared chat data set was merged with patients’ sociodemographic and medical information as well as physician characteristics. Subsequently, the data file was pseudonymised a second time, i.e. all text files received a random generated byte string label that had no connection to the study ID. Within the institute, standard security measurements are in place to restrict access to all computers containing study data. Researchers have no access to the practices’ master files containing patient names and study IDs, which are kept in the participating practices only. All participants with at least one chat message were analysed. The chat analysis was conducted with two data sets: the population of all patients who sent or received at least one chat message (*n* = 363), the subpopulation of the patients who sent or received at least one chat message and completed the final BP measurement at follow-up (*n* = 246 of 363). The latter data set is identical to the patients analysed in the main results paper [19].

#### Data analysis

The chat analyses were performed on patient, practice and physician level with code written in Python 3. Frequencies were calculated for categorical variables, while mean, median, standard deviation (SD) and interquartile range (linear interpolation; IQR) were calculated for continuous variables. Words from word counts were manually grouped into word categories using the word stem and topics. *T*-tests analysed for associations between the Flesch index and physician characteristics: gender, year licenced, practice type (single, group practice), technical affinity (≤ 3, > 3). To account for practice cluster effects in both patient samples, generalised linear mixed models (GLMM) with a significance level of 95% and a Flesch cut-off of 80 (easy language) investigated associations between sociodemographic parameters and patients’ language complexity: age in years (40–49, 50–59, 60–69, 70–79), sex assigned at birth (male, female), education (completed 12th grade, not completed 12th grade) and health status (bad, not good, good, very good, excellent). In addition, a multiple linear regression was estimated to analyse the association between the average number of messages wrote by patients as well as practices per day and the change in patients’ systolic and diastolic blood pressure, standardised for 100 days. In patients who did not reach the target value (< 140/90), we calculated the relative improvement separately for the systolic and diastolic BP: 100*(SBP_baseline − SBP_followup)/(SBP_baseline − 139); 100*(DBP_baseline − DBP_followup)/(DBP_baseline − 89).

The significance level was set at *p* < 0.05. The GLMM was conducted in R 3.6, all other calculations in Python 3.10.

### Role of the funding source

This study was funded by the German Innovation Fund, located at the Federal Joint Committee (Innovationsausschuss beim Gemeinsamen Bundesausschuss, grant number: 01NVF17002). The funder had no role in the study design, data collection and analysis, decision to publish or preparation of the manuscript.

## Results

The data set for analysis comprised 363 patients from the intervention group who sent or received at least one chat message. Of these, 246 patients completed the BP reading at follow-up and sent at least one chat message. Of the 363 patients, 55.9% were male. The average age was 56.5 years, the majority was married or lived with their partner (67.8%), had a good or even better health status (66.1%) and was employed (61.7%). The distribution of characteristics was similar among those with and without a completed follow-up. The participating physicians were 58.3% male, on average had been licenced for 7 years (SD 8.0), and 47.8% worked in a single practice. The average affinity for technology score was 2.8 (SD 1.04) (scale 1 to 6). For details, please see Table 1.

**Table 1:** Sociodemographic and health characteristics of the study population: baseline characteristics of patients, practices and physicians

	**At least one chat**	**At least one chat and follow-up completed**
**Patient characteristics**	***N* = 363**	***N* = 246**
**Sex assigned at birth**, *n* (%)		
Female	160 (44.1)	110 (44.7)
Male	203 (55.9)	136 (55.3)
**Age (in years)**, mean (SD)	56.52 (9.22)	57.49 (8.53)
**Relationship status**, *n* (%)		
Married/living with partner	246 (67.8)	167 (67.9)
Divorced/separated	44 (12.1)	30 (12.2)
Widowed	18 (5.0)	16 (6.5)
Single	42 (11.6)	23 (9.4)
No information	13 (3.6)	10 (4.1)
**Education**, *n* (%)		
Completed 13th grade (Abitur)	70 (19.3)	46 (18.7)
Completed 12th grade	29 (8.0)	19 (7.7)
Completed 10th grade	122 (33.6)	78 (31.7)
Completed 9th grade	106 (29.2)	80 (32.5)
No degree	14 (3.9)	10 (4.1)
Other	7 (1.9)	2 (0.8)
No information	15 (4.1)	11 (4.5)
**Self-rated health status**, *n* (%)		
Excellent	3 (0.8)	1 (0.4)
Very good	36 (9.9)	20 (8.1)
Good	201 (55.4)	138 (56.1)
Not good	97 (26.7)	68 (27.6)
Bad	12 (3.3)	9 (3.7)
No information	14 (3.9)	10 (4.1)
**Professional status**, *n* (%)		
Employed	224 (61.7)	146 (59.4)
Retired/early retirement	87 (24.0)	70 (28.4)
Seeking employment	13 (3.6)	7 (2.9)
Homemaker	10 (2.8)	6 (2.4)
Unemployed	15 (4.1)	7 (2.9)
No information	14 (3.9)	10 (4.1)
**Physician characteristics**	***N* = 24**	***N* = 24**
**Sex assigned at birth**, *n* (%)		
Female	10 (41.7)	10 (41.7)
Male	14 (58.3)	14 (58.3)
**Age (in years)**, mean (SD)	47. 25 (11.04)	47. 25 (11.04)
**Licence to work for the statutory health insurance**, *n* (%)		
2013 or pre-2013	9 (37.5)	9 (37.5)
Post-2013	9 (37.5)	9 (37.5)
No information	6 (25.0)	6 (25.0)
**GPs’ affinity to information technology**, *n* (%)		
Low affinity (scores 1–3)	12 (50.0)	12 (50.0)
High affinity (scores 4–6)	9 (37.5)	9 (37.5)
No information	3 (12.5)	3 (12.5)
**Physicians’ practice type**, *n* (%)		
Single practice	11 (45.8)	11 (45.8)
Group practice	12 (50.0)	12 (50.0)
No information	1 (4.1)	1 (4.1)

### Primary outcome

**Fig. 1:**
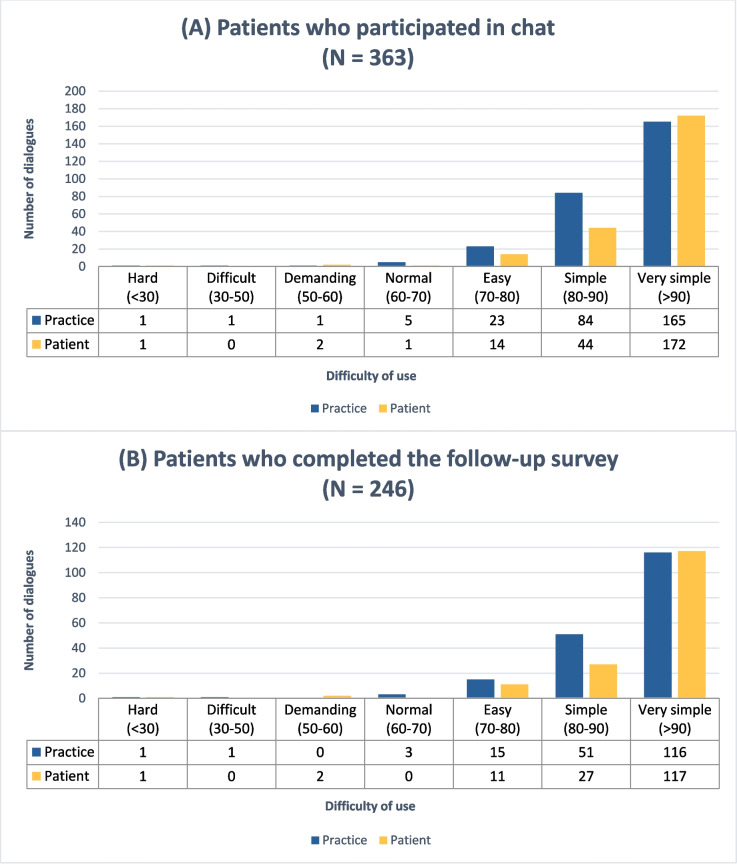
Language difficulty (Flesch values). Difficulty of chat language categorised using the Flesch index

Regarding language complexity, the average Flesch values of the patients’ and practices’ messages showed simple language (patients: 100.1, SD 17.2; practices: 92.6, SD 13.5). Figure 1 shows the distribution with a marked skew towards easy language. Additionally, there was no significant difference in language complexity between the initial and follow-up chats. Across all dialogues, 88.9% of the practices and 92.3% of the patients used simple or very simple language. There was no association between patients’ educational level and the Flesch index of patients and physicians. Also, the patients’ health status had no influence. GPs in single practices used simple and very simple language more frequently than those in group practices without difference by age, gender or number of years in practice. The GPs’ gender (*p* = 0.410), years of practice (*p* = 0.112) and their technical affinity (*p* = 0.056) were not significantly associated with language complexity.

Automated messages to inform patients about a new medication plan or prescription ready for pick up were evaluated separately. These messages showed an average Flesch value of 41.38 (difficult to fairly difficult), with a score of 27.75 for new medication plan (‘Es wurde ein neuer Medikationsplan hochgeladen’) and a score of 55.0 for prescription pick-up (‘Das angeforderte Rezept liegt nun zur Abholung bereit’).

In both statistical models (GLMM), the covariates age, sex assigned at birth, education and health status showed no significant influence on the Flesch index. In the first model (patient with at least one chat), the intra-cluster coefficient (ICC) was 0.16 and the variance of the random effects was 0.63 (SD 1.81). In the second model (patient with at least one chat and follow-up data), the ICC was 0.08 and the variance of the random effects was 0.28 (SD 1.81).

### Secondary outcomes

A total of 4231 messages were sent between practices and patients, including 941 (22.2%) automated messages from practices to inform patients about a new medication plan (*n* = 806) or that a prescription was available for pick-up (*n* = 135). Including the automated messages, the overall ratio of patient-to-practice messages was 31.9% and practice-to-patient messages was 68.1%. All non-automated messages (*n* = 3290) were analysed in detail: 41.0% were sent by patients (*n* = 1349) and 59.0% by practices (*n* = 1941). The mean length of patients’ messages was 22.6 words (SD 22.6) compared to 16.8 (SD 19.4) by practices, and 7.0 (SD 1.41) for automated messages. On average, female physicians send significantly longer messages (100.19 characters) than male physicians (82.40 characters) (*p* < 0.001). The average chat dialogue interval (time between the first message and the last message) was 176.8 days (SD 9.8). On average, a message was sent every 13.2 days (SD 25.1). Of the messages, 33.9% started with a salutation and 37.3% ended with a goodbye phrase. A total of 465 messages contained question marks and were manually categorised. Practices sent 160 explicit questions, 56 requests and 17 combined requests and questions, while the reason for the question mark was unclear in 7 messages. Patients sent 175 explicit questions, 21 requests and 9 combined requests and questions, while there were 20 messages with unclear meaning of the question marks. The chat characteristics of the patients with and without a completed follow-up were similar. The details are presented in Table 2.

**Table 2:** Chat characteristics

	**Patients with at least one chat (*N* = 363)**	**Patients with at least one chat and follow-up completed (*N* = 246)**
**Messages**		
Messages sent, *n* (%)	4231 (100%)	2800 (100%)
Patients who sent at least 1 message, *n* (%)	234 (64.5%)	158 (64.2%)
Patients who received at least 1 message from their practice, *n* (%)	358 (98.6%)	244 (99.2%)
Patients who received at least 1 non-automated message from their practice, *n* (%)	280 (77.1%)	187 (76.0%)
*Practice to patient*	2882 (68.1%)	1831 (65.2%)
Not automatically generated, *n* (%)	1941 (45.9%)	1179 (42.1%)
Automatically generated, *n* (%)	941 (22.2%)	652 (23.3%)
New medication plan, *n* (%)	806 (19.0%)	550 (19.6%)
Prescription ready for pick-up, *n* (%)	135 (3.2%)	102 (3.6%)
*Patient to practice*	1349 (31.9%)	969 (34.6%)
**Words used in chats**		
Unique words used overall	2020	1723
Salutations	404 (28.2%)	253 (27.3%)
Salutations per patient, mean (SD)	2.4 (9.2)	2.0 (3.5)
Goodbye phrases	369 (23.4%)	257 (25.2%)
Goodbye phrases per patient, mean (SD)	1.7 (2.6)	1.5 (2.5)
*Practice to patient*		
**Words used in chats**		
Unique words used overall	2810	2170
Salutations	1029 (71.8%)	673 (72.7)
Salutations per patient, mean (SD)	2.8 (5.9)	2.6 (5.6)
Goodbye phrases	1210 (76.6%)	763 (74.8%)
Goodbye phrases per patient, mean (SD)	3.3 (6.6)	3.0 (6.2)
**Frequencies and lengths**		
Number of words practice to patient, median (IQR)	13.0 (24.0)	10.0 (20.0)
Number of characters practice to patient, median (IQR)	64.0 (128.0)	60.0 (128.0)
Number of words patient to practice, median (IQR)	16 (23.0)	14.0 (19.0)
Number of characters patient to practice, median (IQR)	79.0 (114.0)	75.0 (115.0)
Duration of conversation (min 1 each) (days), median (IQR)	175.2 (148.4)	168.5 (151.7)
Time period between messages by practice (days), median (IQR)	8.2 (18.1)	9.2 (18.3)
Time period between messages by patient (days), median (IQR)	2.4 (19.0)	2.2 (17.7)
Number of unique words used per patient, median (IQR)	51.0 (110.0)	50.5 (106.25)
Number of unique words used per message, median (IQR)	12.0 (19.0)	11.0 (19.0)
**Subjective well-being and time interval of patients chats to practices**		
Subjective well-being (indicated by one of 5 types of smileys when reporting blood pressure), median (IQR)	4.0 (1.0)	4.0 (1.0)

The grouping of words in the non-automated messages (*n* = 363) showed the following key topics in decreasing order: 1. blood pressure, 2. measurements, 3. pharmacological agents/medication brand names, 4. medication, 5. pills, 6. prescriptions. Our study found significant differences in words used by patients and practices. Patients used general health terms, while practices focused on technical and treatment-related language. Chi-square tests confirmed these differences (e.g. blood pressure *p* < 0.001, measurements *p* < 0.001). For further details, see Table 3.

**Table 3:** Chat topic analysis (*n* = 363)

Word category	Most frequently used word variation (in German)	Total number of occurrences		Number of occurrences by patients		Number of occurrences by practice		*p* values
		***n***	**%**	***n***	**%**	***n***	**%**	
Total number of words		*64,164*	*100.00*	27,545	*42.93*	*36,619*	*57.07*	*1.0*
Blood pressure	Blutdruck Blutdruckwerte RR	1110	1.73	146	13.15	964	86.85	< 0.001
Measurements	Messen Messungen Gemessen	724	1.13	176	24.31	548	75.69	< 0.001
Pharmacological agents/medication brand names	Ramipril Amlodipin Candesartan	619	0.10	298	48.14	321	51.86	0.008
Medication	Medikamente Medikament Med	528	0.8	172	32.58	356	67.42	1.53
Pills	Tablette Tabletten Tabl	246	0.38	130	52.85	116	47.15	0.001
Prescription	Rezept Rezepte Rezeptbestellung	227	0.35	100	44.00	127	55.90	0.73
Practice	Praxis Praxisteam	222	0.35	59	26.58	163	73.42	< 0.001
Appointment	Termin Impftermin Labortermin	118	0.18	74	62.71	44	37.29	< 0.001
Eating and drinking	Wasser (drinking-related ones only) Bier Kaffee	98	0.15	69	70.41	29	29.59	< 0.001
Therapy	Therapie Therapieänderung Therapieanpassung	90	0.14	5	5.56	85	94.44	< 0.001
Vaccination	Impfung Geimpft Impftermin	89	0.14	56	62.92	33	37.08	< 0.001
Oxygen	Sauerstoff Sauerstoffsättigung	64	0.10	63	98.44	1	1.56	< 0.001
Phone	Telefonisch Telefon Telefonieren	63	0.10	25	39.68	38	60.32	0.60
Pulse	Puls Pulswerte Ruhepuls	63	0.10	42	66.67	21	33.33	0.0001
Pain	Kopfschmerzen Schmerzen Schmerzt	61	0.10	52	85.25	9	14.75	< 0.001
Vacation	Urlaub Urlaubstage	52	0.08	22	42.31	30	57.69	0.92
Stress	Stress Stressige Stresssituationen	52	0.08	32	61.54	20	38.46	0.006
Water	Wasser Wassertablette Wassertabl	47	0.07	35	74.47	12	25.53	< 0.001
Sport and exercise	Sport Bewegung Fußball	25	0.04	16	64.00	9	36.00	0.03
Diabetes	Blutzucker Diabetes Blutzuckerwerten	9	0.01	6	66.67	3	33.33	0.15

The chat content analysis revealed differences in the frequency of topics in the communication between GPs and patients. ‘Update on BP treatment, measurements, medication’ was the most prevalent topic, accounting for 915 (47.1%) instances. This category was frequently associated with ‘Messages on further procedures’, which occurred 312 times (16.1%). Additionally, ‘Request for (and inquiries about) BP measurements’ and ‘BP medication change’ were notable categories, with 355 (18.3%) and 233 (12.0%) instances, respectively. The analysis of patient-to-practice communication revealed that ‘Confirmation or thanks’, with 312 (23.2%) instances, emerged as the most prevalent category. ‘Other medication and treatments’ emerged as a significant category, with 181 (13.4%) instances; however, one third of this category is attributed to a single patient sharing his oxygen saturation readings. Moreover, ‘BP medication’ remained a prominent category, with 156 (11.6%) instances, indicating a continued focus on BP medication management in patient-initiated communication. This analysis by topic showed that BP measurements (18.29% vs. 5.04%; *p* = 0.03), study proceedings (19.70% vs. 10.46%; *p* = 0.01) and inquiries on further procedures (16.07% vs. 6.90%; *p* = 0.01) were more frequently addressed by practices than patients, emphasising the focus on treatment guidance, monitoring and follow-up in hypertension management. Details are provided in Table 4.

**Table 4:** Chat messages content analysis

	**Practice to patient (*N* = 1941)**		**Patient to practice (*N* = 1348)**		***p* value**
	***n***	**%**	***n***	**%**	
*Medication or treatments*	*1726*	*88.92*	*700*	*51.93*	< *0.001*
BP measurements	355	18.29	68	5.04	*0.003*
BP medication	233	12.00	156	11.57	*1.0*
In office consultation	60	3.09	14	1.04	*1.0*
Inquires about vital signs, symptoms, problems or diseases	20	1.03	158	11.72	*0.1*
*Study proceedings*	*340*	*19.70*	*141*	*10.46*	*0.01*
Inquiries on further procedures	312	16.07	93	6.90	*0.01*
(Chat) technology	28	1.44	48	3.56	*0.52*
*Administration*	*77*	*4.46*	*161*	*11.94*	*0.05*
Scheduling request	38	1.96	56	4.15	*1.0*
Phone call request	14	0.72	14	1.04	*1.0*
*Risk factors*	*17*	*0.98*	*83*	*6.16*	*0.58*
Nutrition	10	0.52	16	1.19	*1.0*
Psychosocial problems	3	0.15	24	1.78	*1.0*
Life issues	3	0.15	23	1.71	*1.0*
Physical activity	1	0.05	20	1.48	*1.0*
*Others*	*213*	*12.34*	*496*	*36.80*	< *0.001*
Confirmation or thanks	150	7.73	312	23.15	< *0.001*
Other	63	3.25	60	4.45	*0.67*
Inquiries about patient’s well-being	87	4.48	–	–	–
Disclose BP	–	–	100	7.42	–
Update on other treatment and medication	46	2.37	–	–	–
Prescription change	–	–	23	1.71	–
Measures against other symptoms, problems or diseases	10	0.52	–	–	–
Update on BP treatment, measurements, medication	915	47.14	–	–	–
Other medication and treatments	–	–	181	13.43	–
Prescription refill	–	–	84	6.23	–
BP medication refill	25	1.29	–	–	–
Referral request	–	–	7	0.52	–
Other notification	–	–	124	9.20	–

Categories differ slightly between the practice to patient messages and patient to practice messages

The linear regression model showed that more frequent chat messages from practices to patients (SBP *β* = 1.63; *p* < 0.001; DBP *β* = 1.69, *p* < 0.001) and from patients to practices (SBP *β* = 9.06, *p* < 0.001; DBP *β* = 10.08, *p* < 0.001) were significantly associated with better BP control. For further details, see Table 5.

**Table 5:** Relationship between chat message frequency (practice-to-patient and patient-to-practice) and SBP/DBP control (linear regression model)

Variable	Coefficient (*β*)	*T* value	*p* value	95% CI
Message from patients (SBP)	9.06	6.80	< 0.001	[6.44, 11.69]
Message from practices (SBP)	1.63	4.28	< 0.001	[0.88, 2.37]
Message from patients (DBP)	10.08	7.62	< 0.001	[7.48, 12.69]
Message from practices (DBP)	1.69	4.49	< 0.001	[0.95, 2.44]

**Fig. 2:**
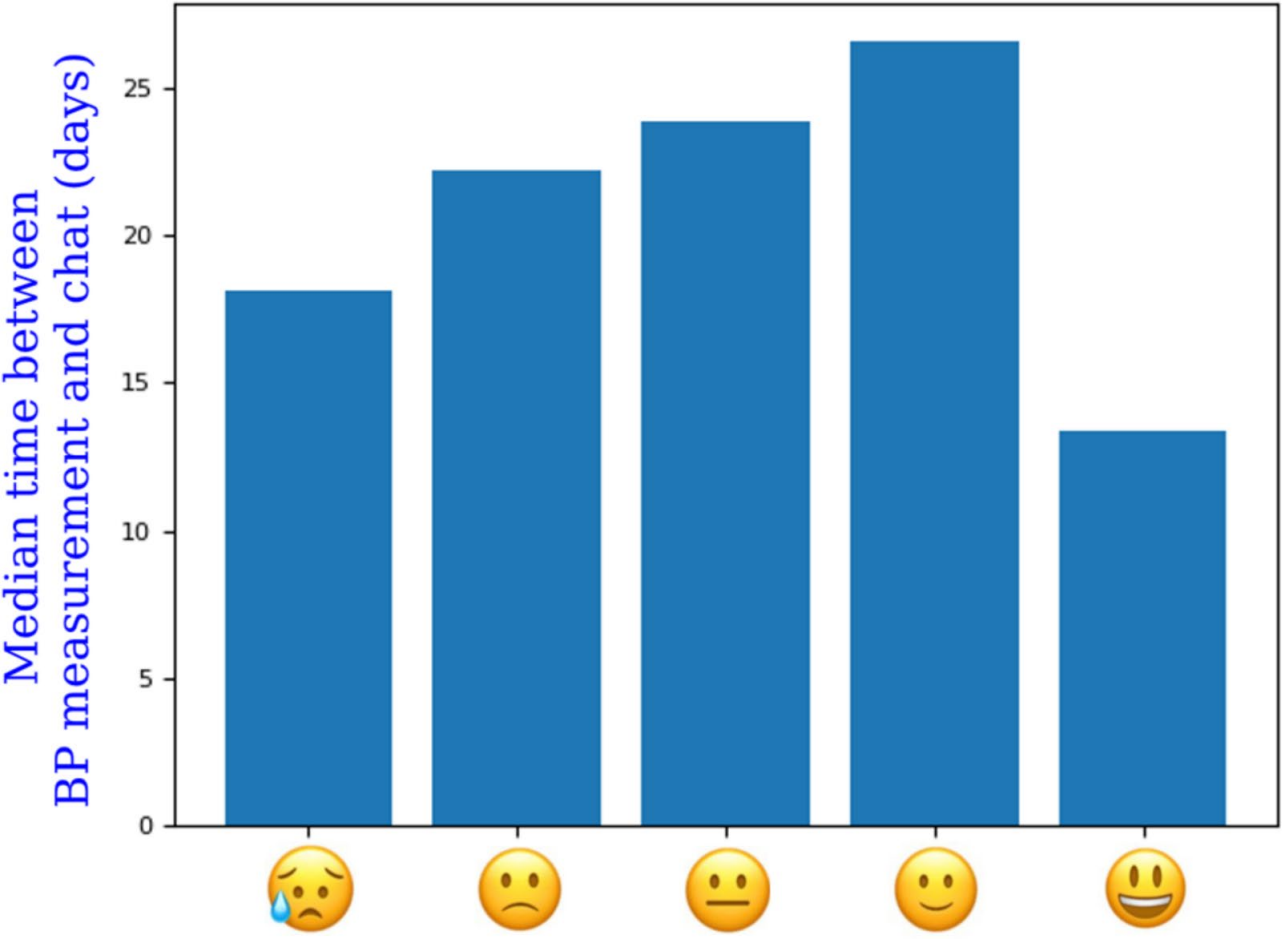
Correlation between patients’ perceived well-being and the time to a message. This figure illustrates the correlation between patients’ subjective well-being (represented by the smileys, which patients selected in the app when performing a blood pressure (BP) measurement) and the time between the last BP measurements before a message to the practitioner

Patients’ subjective average health was 3.66 (SD 0.76) over a total of 43,365 measurements. Figure 2 shows the association between patients’ subjective well-being and the time interval to the next chat message. When patients perceived their well-being two points lower than their individual mean (*n* = 48), they contacted their GP more than 15 days earlier on average. Even 0.1 point lower than the mean reduced the time interval by more than 3 days (*n* = 234). There were no adverse events.

Additional observations are noteworthy. First, to detect potential user or software problems, all messages containing the word ‘app’ or ‘smartphone’ were reviewed by the first author. The word ‘app’ was used 94 times, ‘handy/cell phone/smartphone’ 15 times and ‘program’ 10 times. Two patients needed to reactivate the app because of a lost or new smartphone. Physicians frequently used the word ‘app’ to encourage patients to perform and transmit more blood pressure readings via the app. All mentions of the word ‘telephone’ were manually reviewed. There were 11 call requests by patients and 17 by practices. Practices also often offered a phone call in farewell clauses (*n* = 11) while patients mentioned this in only 3 cases.

Second, the chat communication was used to exchange a variety of information beyond hypertension. For example, a recent tragic event (severe regional flooding) was mentioned 8 times. Given the ongoing national vaccination campaign against SARS-CoV-2 at the time, issues related to vaccinations were mentioned 89 times. Other medical topics were also addressed: ‘Sauerstoff’ (oxygen) was mentioned 64 times by a single patient reporting his oxygen saturation. Type 2 diabetes treatment (metformin: 9 times), hypercholesterolaemia (atorvastatin: 7 times) and inflammatory bowel disease (sulfasalazine: 6 times) were also mentioned. The app was also used to contact physicians for issues relating to pain (61 counts) and nausea (7 counts).

Third, according to the inclusion criteria, the PIA study included patients with hypertension in whom lifestyle measures alone were not sufficient and who needed at least one medication. Healthy living and lifestyle changes were mentioned a few times in the form of eating, drinking, sports, sleeping and stress. Even unhealthy beverages like beer were mentioned by the patients. However, there were more mentions of water (*n* = 35): 30 were related to how much water they drank during the day and 5 were related to leg swelling. The physicians answered with increases in diuretics and/or change of amlodipine medication via the PIA system.

## Discussion

This analysis of the PIA chat communication showed that the app-based messaging system was frequently used and widely accepted by patients and physicians. Even more interesting: the complexity of patients’ and physicians’ language as measured by the Flesch index was rated as easy, simple and very simple, with no differences based on patients’ health status, educational degree or physician characteristics. This result refutes the widespread notion that physicians tend to use difficult language that impairs patients’ understanding. However, as this study only analysed data from GPs, these findings may not be generalisable to all types of physicians. Several approaches to analyse language complexity are published in the literature, ranging from mathematical calculations like the Flesch index to recent approaches using neural networks. In our study, the majority of the language used was simple, as graded by the Flesch index. Interestingly, neither patients’ education, age or sex nor physicians’ age showed any differences. It is noteworthy that there was no specific training in simple language for the physicians. However, they were licenced primary care specialists for an average of 19 years. This suggests that their professional experience, combined with the chat format, supported the use of simple language on behalf of the physicians. While the Flesch index is the most widely used approach for classifying language complexity, other approaches are emerging with automated data analysis. In 2012, Hancke developed a neural network model to categorise language difficulty for the German language [29]. Using a freely available artificial intelligence (AI) platform, the language complexity of various orthopaedic patient education materials on herniated lumbar discs, scoliosis and lumbar stenosis decreased from a Flesch-Kincaid grade level of 9.5 to 12.6 to a level of 5.0 to 6.9 [30]. In the future, comparisons between traditional readability measures and AI-supported approaches will be of interest. Our findings indicate that automated messages had a significantly higher language complexity compared to non-automated messages. AI-driven simplification, as discussed by Bernstein [30] in the context of patient education materials, could similarly enhance the readability of automated messages in clinical communication. Although it is well-known that the use of easy language is highly relevant to patients and public health, its application across all areas remains a work in progress. The support by public institutions such as the European Commission and other public agencies show its importance.

In addition, this study showed that the mean length of patients’ messages was longer, compared messages from practices. This finding is in line with previous research showing that patient emails to clinicians are typically longer than clinician responses [31]. Similarly, this study found gender differences in message length, with female physicians writing longer messages than male physicians, consistent with prior research on gender differences in medical communication [32–34]. However, this study did not find an association between physician gender and language complexity, which is consistent with prior research showing that while female physicians engage more in patient-centred communication, they do not differ from male physicians in the provision of biomedical information or overall message complexity [35].

In recent years, video consultations and personal chat messaging have changed the practice of primary care. Yet, the interplay of in-person consultations, phone contacts, online visits, letters, email and messaging is still evolving. J.K. Fujioka et al. conducted a qualitative study with 26 practitioners in Southern Ontario and found that virtual visits can effectively resolve many patient concerns. However, physicians were concerned that some patients might overuse the virtual system due to its convenience and flexibility or use the system inappropriately due to limitations in their technical capabilities. The GPs preferred asynchronous messaging to virtual visits, as this allows more flexible time management. Non-virtual approaches were preferred not only when physical examination or urgent care is required but also for prescribing narcotics, first visits of new patients or for patients with severe mental health issues [36]. Similarly, in our study, the GPs highly valued the asynchronous messaging system but initiated additional phone calls or practice visits in more complex situations.

Previous studies analysing chat content between physicians have identified five main meta-categories: medication or treatments, study proceedings, risk factors, administration and other. Some studies found a significant focus on administrative requests in physician communication [25, 26]. Other studies documented a substantial number of messages related to medication or treatments [22, 24]. Similarly, the present study based on chat data from the PIA study observed a considerable amount of communication between patients and practices regarding hypertension management and its associated medication treatment. Also, more frequent chat messages were significantly associated with better BP control. This underscores how chat infrastructure not only aids in simplifying administrative processes, but also contributes to improving patient treatment.

Regarding strengths and limitations, this study examined a large number of chat dialogues and represents one of the few existing scientific analyses of chat dialogues within a primary care setting. The data represent patients from various age groups and educational backgrounds. The well-established Flesch index for the German language allowed for a comparison with other studies. Although language complexity is known to interplay with patients’ understanding of medical information, we did not measure patients’ comprehension of the information provided by practices. We acknowledge that patients’ awareness of the study may have influenced their language use. Also, we did not assess patients’ familiarity with hypertension-related language or how their experience with the condition influences their communication. We acknowledge that access to smartphones and the necessary digital literacy may limit the generalisability of our findings, particularly across different countries. The study focused on hypertension control and may not necessarily be applicable to other patient groups, diseases and settings.

## Conclusions

The secure PIA chat was used frequently by patients and physicians alike. Because data protection regulations prohibit the use of chat applications like WhatsApp or Signal for routine patient care, such a chat messaging system is ground-breaking for Germany. Our data support the use of chat communication for topics such as hypertension care in primary care. Future research on language complexity in artificial intelligence-generated chat communication and more diverse patient populations is needed.

## Data Availability

No datasets were generated or analysed during the current study.

## Abbreviations

*β*: Beta coefficient
BP: Blood pressure
ICC: Inter-cluster coefficient
ICT: Information communication technology
IQR: Interquartile range
GLMM: Generalised linear mixed model
GP: General practitioner
PIA: PC-gestütztes Fallmanagement von Hypertonikern zur Implementierung einer Leitlinien-konformen Hypertonietherapie anhand eines Arzt-definierten und – supervidierten, patientenindividuellen Therapiealgorithmus (= official German title which stands for ‘PC-supported case management of hypertensive patients to implement guideline-based hypertension therapy using a physician-defined and -supervised, patient-specific therapeutic algorithm’)
PIA-PrMC: PIA practice management centre
SD: Standard deviation
